# Use of MRP8/14 in clinical practice as a predictor of outcome after methotrexate withdrawal in patients with juvenile idiopathic arthritis

**DOI:** 10.1007/s10067-022-06165-4

**Published:** 2022-04-29

**Authors:** Emma J Welsh, Beverley Almeida, Jason Palman, Peter Bale, Clare Heard, Dirk Holzinger, Johannes Roth, Dirk Foell, Emily Robinson, Simona Ursu, Chris Wallace, Kimberly Gilmour, Lucy R. Wedderburn, Elizabeth Ralph

**Affiliations:** 1 Infection Inflammation and Rheumatology Department, University College London, Great Ormond Street Institute of Child Health, 30 Guilford Street, London, WC1N 1EH UK; 2 NIHR BRC, Great Ormond Street Hospital for Children NHS Trust, London, UK; 3 Department of Rheumatology, Great Ormond Street Hospital for Children NHS Trust, London, UK; 4 Department of Paediatric Haemato-Oncology, University of Duisburg-Essen, Essen, Germany; 5 Institute of Immunology, University of Munster, Munster, Germany; 6 Department of Paediatric Rheumatology and Immunology, University Hospital Munster, Munster, Germany; 7 MRC Biostatistics Unit, Cambridge Biomedical Campus, Cambridge Institute of Public Health, University of Cambridge, Cambridge, UK; 8 Cambridge Institute of Therapeutic Immunology & Infectious Disease (CITIID), Department of Medicine, Jeffrey Cheah Biomedical Centre, Cambridge Biomedical Campus, University of Cambridge, Cambridge, UK; 9 Department of Immunology, Great Ormond Street Hospital for Children NHS Trust, London, UK; 10 Centre for Adolescent Rheumatology Versus Arthritis at UCL, UCLH & GOSH, London, UK

**Keywords:** Juvenile idiopathic arthritis, Methotrexate, Outcomes research

## Abstract

The objective of this study was to determine the effectiveness of MRP8/14 as a predictor of disease flare in patients with juvenile idiopathic arthritis (JIA) following the withdrawal of methotrexate (MTX) in a routine clinical setting. All MRP8/14 tests performed at a single centre in a 27-month period were considered for analysis. Patients were assessed against criteria for inactive disease and subsequent disease flare. Decisions on whether or not to stop treatment were recorded. MRP8/14 results were assessed in conjunction with clinical information. Clinicians were also surveyed to investigate if MRP8/14 influenced their decision to discontinue MTX where this was available at that time point. One hundred four cases met the inclusion criteria during the study period. Although there was no significant difference in flares between patients with an elevated or low MRP8/14 value, in those who stopped MTX (*n* = 22), no patients with a low MRP8/14 (≤ 4000 ng/ml) result flared (follow-up time 12 months). Clinicians reported that for patients with clinically inactive disease and an elevated MRP8/14 result (> 4000 ng/ml), none would advise withdrawal of MTX. Low MRP8/14 was interpreted favourably when considering stopping MTX treatment in patients with JIA. Implementation of MRP8/14 testing has changed clinical practice at this centre. However, the observation that some patients in our cohort who had an elevated MRP8/14 value did not flare after stopping MTX for non-disease-related reasons highlights the need for further biomarkers to predict the risk of flare off medication in JIA and aid clinicians in treatment decisions.

**Table:** 

**Key Points** *• First study of serum MRP8/14 measurement in clinical practice to inform treatment decisions in patients with JIA.* *• No patients with a low MRP8/14 test result went on to suffer a disease flare in 12 months of follow follow-up.* *• Further biomarkers are needed to predict the risk of flare off medication in JIA and treatment decisions.*

**Supplementary Information:**

The online version contains supplementary material available at 10.1007/s10067-022-06165-4.

## Introduction

Juvenile idiopathic arthritis (JIA) is a paediatric chronic autoimmune inflammatory condition characterised by joint swelling and pain. Methotrexate (MTX) is the most widely used disease-modifying anti-rheumatic drug (DMARD) and is used as a first-line systemic treatment for JIA. Although many patients achieve disease remission with DMARDs, the definition of this remains difficult. Fifty percent of patients who appear to be in clinical remission as defined by previously published criteria [1, 2] will experience a disease flare after discontinuing MTX [3].

Patients would benefit from a sensitive biomarker that could identify subclinical inflammation and accurately predict the risk of flare if MTX were stopped, allowing clinicians to make a more informed decision on whether to alter patient medication. It has been previously suggested that S100 serum protein concentrations correlate with inflammation in JIA [4, 5]. Two proteins from this family, myeloid-related protein 8 (MRP8, S100A8) and MRP14 (S100A9), form a heterodimer known as calprotectin or MRP8/14. Both proteins are highly expressed and released at local sites of inflammation and play a functional role in the process of arthritis [6, 7]. Higher serum concentrations of this protein complex prior to MTX discontinuation have been shown in an international trial to be associated with the risk of disease flare after discontinuing MTX [8], as well as with the good clinical response to MTX when measured prior to treatment [9].

In this study, we assessed the use of MRP8/14 in clinical practice at one centre to evaluate its effectiveness as a predictor of flare in the 12 months following withdrawal of MTX.

## Methods

### Patients

All MRP8/14 tests performed at a single-paediatric rheumatology centre in a 27-month period were considered for analysis. Patients selected for this analysis were those with any subtype of JIA [10] who were on MTX monotherapy for their arthritis at the time of the test, had not previously been on a biologic or other systemic disease-modifying drugs, and had the test for consideration of stopping MTX. Where multiple tests were performed on the same patient over time, only the first test was used for analysis. Clinically inactive disease (CID) was defined for each patient using Wallace criteria [2]—no active joints, no systemic features (fever, rash, lymphadenopathy, serositis, splenomegaly), no uveitis, normal erythrocyte sedimentation rate (ESR) and C-reactive protein (CRP) levels, and no disease activity as rated by physician’s global assessment. JIA subtype, gender, age at diagnosis, disease duration, and age at sample were recorded. Data on ethnicity was recorded and patients categorised as Caucasian, other or not specified. Ethnicity data was self-reported from patients and their families.

Disease flares in a period of 12 months after MTX withdrawal in patients off MTX were defined as the development of arthritis in one or more joints that required new intervention (restarting MTX, oral or intravenous steroid treatment, or intra-articular joint injections). A survey of the use of the MRP8/14 test was also circulated to senior members (Supplementary Table S1) of the paediatric Rheumatology team.

### Measurement of MRP8/14

Testing for MRP8/14 was established at our centre for clinical use. MRP8/14 assay detailed methods are found in Supplementary Material and Figure S1. In brief, blood samples for serum MRP8/14 concentration were collected from patients at routine clinic appointments when clinicians were considering stopping MTX treatment. Samples were centrifuged at 850 g for 10 min, and serum was stored at – 70 °C until assayed by sandwich enzyme-linked immunosorbent assay (ELISA) (Buhlmann Laboratories AG, Switzerland), following the manufacturer’s instructions with the modification that samples were assayed at a 1:400 dilution as well as 1:100 dilution. The final concentration of MRP8/14 was calculated by averaging the results obtained at 1:100 and 1:400 (Biotek ELx808 absorbance microplate reader, Biotek Instruments Inc., USA, using Gen5 Reader control software). Cutoff levels for MRP8/14 measurements for clinical use (including a threshold for what was defined as low MRP8/14 concentration) were determined by extrapolation from published data, see Supplementary Material.

### Statistical analysis

The majority of statistical analyses were performed using Prism Graphpad Version 7.0. Results were not normally distributed so statistical analyses used non-parametric tests. Demographics were summarised using descriptive statistics; median and interquartile ranges were reported where appropriate. Demographic groups were analysed using a one-way ANOVA (Kruskal–Wallis test) and Dunn’s multiple comparisons tests. Comparison of MRP8/14 results between groups was by the Mann–Whitney test. Flares over time were plotted as a Kaplan–Meier curve; groups were compared using a log-rank (Mantel-Cox) test. Deep analysis of MRP8/14 value association with the prediction of flare was performed using cox proportional hazards, carried out using R version 3.5.1 (see Supplementary Material for script).

## Results

### Patients and demographics

**Fig. 1:**
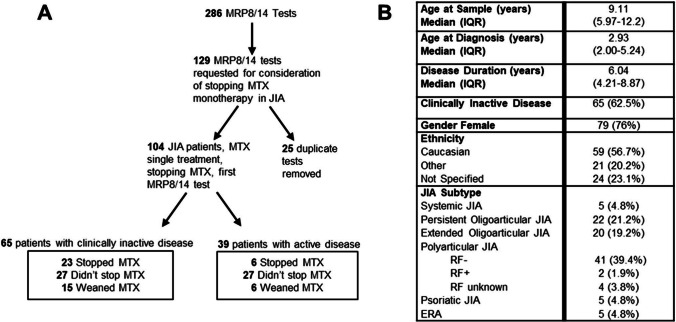
**A** Flow of tests in patients for whom stopping methotrexate was considered. Note: Clinically inactive disease defined by Wallace criteria [1]. **B** Demographic and clinical data of patients who met the criteria for inclusion in this analysis (*n* = 104). Note: 25 duplicate tests from 22 patients

Two hundred eighty-six MRP8/14 tests were requested at a single-paediatric rheumatology centre over the study period. One hundred fifty-seven tests were excluded from the analysis as they did not meet the selection criteria for this analysis. Reasons for exclusion were patients without a diagnosis of JIA, those who were on treatment with additional DMARD (other than MTX) or biologic therapy, or those who were having the test for a different reason other than consideration of stopping MTX treatment. Nineteen patients each had two tests and three patients had three tests; the first tests from these patients were used in the final analysis (25 tests excluded) (Fig. 1A). One hundred four tests from 104 patients met the selection criteria and these cases were divided by those with clinically inactive disease (CID, defined by Wallace criteria [2]) and whether they consequently stopped, weaned, or did not stop MTX.

Patient demographic and clinical data are shown in Fig. 1B. The majority of the cohort was female, in keeping with the known prevalence of JIA [11]. Oligoarticular and polyarticular (rheumatoid factor negative) forms of JIA represented the majority of the cohort (79.8%). There was no statistical significance in demographic data between those who reached CID and those who did not.

### Influence of MRP8/14 testing on clinical decision-making

Five senior paediatric rheumatology clinical staff responded to a survey about clinical decision-making and MRP8/14 testing. Clinicians reported that MRP8/14 tests were ordered for stopping, starting, or escalating medication, checking the response to therapy, and assessing for a flare of the disease. In patients on MTX only who had a low serum MRP8/14 result (≤ 4000 ng/ml) and had reached clinically inactive disease, all staff would consider stopping MTX as an initial de-escalation. For patients in CID that had an elevated MRP8/14 result (> 4000 ng/ml), no staff reported that they would consider advising the withdrawal of MTX (Supplementary Table S1).

MRP8/14 results included in the analysis were recorded during the implementation phase of MRP8/14 as a clinical test. Some clinicians did not have the MRP8/14 test result available to them in the clinic at the time of the decision to stop or to continue MTX due to batches of tests performed during the early period of test introduction. The turnaround time of MRP8/14 result availability improved during the time of this clinical audit. Reasons for stopping MTX in a patient whose MRP8/14 value were considered “elevated” included drug intolerance and clinician decision.

### Analysis of flares in patients who reached* CID*

**Fig. 2:**
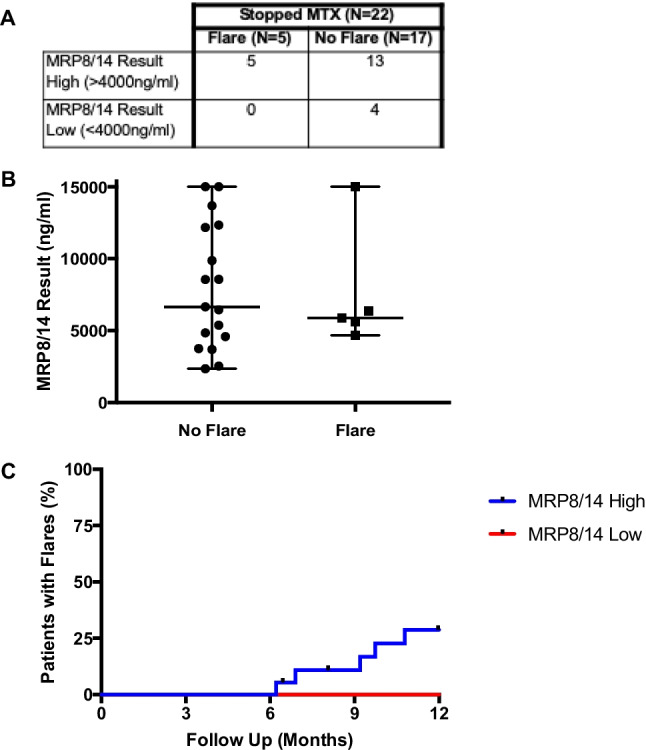
**A** Flares in patients with the clinically inactive disease who stopped methotrexate included in the analysis, divided by MRP8/14 result. **B** Comparison of MRP8/14 test values in patients with the clinically inactive disease who stopped methotrexate divided by flare or no flare following treatment withdrawal (*n* = 22), *p*-value > 0.05. C. Flares after withdrawal of methotrexate (MTX) in patients with clinically inactive disease (*n* = 22) within 12 months of stopping, *p*-value > 0.05. Note: one patient was excluded as lost to follow-up. MTX, methotrexate; MRP, myeloid-related protein; ng, nanograms; ml, millilitres

Flares in patients who achieved CID and stopped MTX are shown split by MRP8/14 result (Fig. 2A). In those who stopped MTX (*n* = 22 one patient lost to follow-up), none of the four patients with a low MRP8/14 result flared in the 12-month period, giving a sensitivity of 100%, specificity of 23.5%, and positive predictive value of 27% in this small group. Raw MRP8/14 values are shown in Fig. 2B.

Flares in patients on MTX only, who both met Wallace criteria (in respect to clinically inactive disease) and stopped MTX treatment were plotted over 12 months (Fig. 2C), split by MRP8/14 result value low (≤ 4000 ng/ml) and elevated (> 4000 ng/ml). Statistical analysis showed no significant difference (*p*-value 0.32) in flares between groups.

Statistical analysis of the MRP8/14 results was performed using R to assess MRP8/14 as a continuous variable using Cox proportional hazards. Despite hazard ratios suggesting low MRP8/14 value may be associated with less flare, low sample sizes mean the estimate has a large degree of uncertainty (high standard error), and therefore, from this study, we cannot be confident that low MRP8/14 would be predictive of less flare in further patients.

## Discussion

This is the first study of MRP8/14 serum measurement in clinical practice to inform treatment decisions in patients with JIA. No patient with a low MRP8/14 who had achieved CID prior to MTX withdrawal flared within 12 months of follow-up. While the comparison between MRP8/14 values and flares over 12 months showed no significant difference, likely due to small sample size, this result mirrors trends previously described in the literature [8]. Clear limitations of the study are the small numbers of cases who met all the criteria for the comparison and the fact that early on in assay implementation the result turnaround time was relatively long, making it more difficult for clinicians to incorporate the result into decision-making with patients and their families.

Our survey of clinical staff showed that the MRP8/14 result is viewed favourably by clinicians to influence their decision to stop treatment; hence, there were no patients in the “did not stop” MTX group with a low MRP8/14. The finding that clinicians reported that for patients with clinically inactive disease and an elevated MRP8/14 result (> 4000 ng/ml), none would advise withdrawal of MTX, and the observation that some patients in our cohort who had an elevated MRP8/14 value did not flare after stopping MTX for non-disease-related reasons (e.g., drug intolerance) highlights the need for further biomarkers to predict the risk of flare off medication in JIA and aid clinicians in treatment decisions.

## Conclusion

In those who stopped MTX, no patients with a low MRP8/14 result flared during the follow-up period. As such, low MRP8/14 may be interpreted favourably by clinicians when considering stopping MTX treatment in patients with JIA. While MRP8/14 often influenced the decision of clinicians to discontinue MTX, it should be noted that there was no significant difference in flares between patients with an elevated or low MRP8/14 value, highlighting the need for further biomarkers to predict the risk of flare.

## Supplementary Information

Below is the link to the electronic supplementary material.

Supplementary file1 (DOCX 82.5 KB)

## Data Availability

All data relevant to the study are included in the article or uploaded as supplementary information. Further information can be provided on reasonable request (and provided if ethically permitted) via the corresponding author if required.
