# Correction: Use of MRP8/14 in clinical practice as a predictor of outcome after methotrexate withdrawal in patients with juvenile idiopathic arthritis

**DOI:** 10.1007/s10067-025-07845-7

**Published:** 2025-12-11

**Authors:** Emma J Welsh, Beverley Almeida, Jason Palman, Peter Bale, Clare Heard, Dirk Holzinger, Johannes Roth, Dirk Foell, Emily Robinson, Simona Ursu, Chris Wallace, Kimberly Gilmour, Lucy R. Wedderburn, Elizabeth Ralph

**Affiliations:** 1 Infection Inflammation and Rheumatology Department, University College London, Great Ormond Street Institute of Child Health, 30 Guilford Street, London, WC1N 1EH UK; 2 NIHR BRC, Great Ormond Street Hospital for Children NHS Trust, London, UK; 3 Department of Rheumatology, Great Ormond Street Hospital for Children NHS Trust, London, UK; 4 Department of Paediatric Haemato-Oncology, University of Duisburg-Essen, Essen, Germany; 5 Institute of Immunology, University of Munster, Munster, Germany; 6 Department of Paediatric Rheumatology and Immunology, University Hospital Munster, Munster, Germany; 7 MRC Biostatistics Unit, Cambridge Biomedical Campus, Cambridge Institute of Public Health, University of Cambridge, Cambridge, UK; 8 Cambridge Institute of Therapeutic Immunology & Infectious Disease (CITIID), Department of Medicine, Jeffrey Cheah Biomedical Centre, Cambridge Biomedical Campus, University of Cambridge, Cambridge, UK; 9 Department of Immunology, Great Ormond Street Hospital for Children NHS Trust, London, UK; 10 Centre for Adolescent Rheumatology Versus Arthritis at UCL, UCLH & GOSH, London, UK

**Correction: Clinical Rheumatology (2022) 41:2825-2830**

10.1007/s10067-022-06165-4

In this article the author’s name "Emma J. Sumner" must be updated to "Emma J Welsh" following marriage surname.

The original article has been corrected.

