# Supplementary material for: Use of MRP8/14 in clinical practice as a predictor of outcome after Methotrexate withdrawal in patients with Juvenile Idiopathic Arthritis
Source: Clin Rheumatol. Author manuscript; Available in PMC 2022 Sep 23. (PMC9474586; doi:10.1007/s10067-022-06165-4)
Supplement: Supplementary Information [file EMS144705-supplement-Supplementary_Information.pdf]

SUPPLEMENTARY INFORMATION

Establishment and Accreditation of MRP8/14 Testing

The assay for measuring MRP8/14 was validated in accordance with the International Standards for Medical Laboratories, ISO 15189 so that the assay could be accredited by the United Kingdom Accreditation Service (UKAS) for clinical use. A number of criteria were established and assay performance against these was validated. This included inter- and intra-assay variability, linearity of the assay, sample stability, and determination of whether a paediatric reference range was required.

The ELISA method is as follows: a monoclonal capture antibody (mAb) highly specific to the MRP8/14 heterodimeric and polymeric complexes is coated onto the microtiter plate. Serum samples are diluted at 1:100 and 1:400 with incubation buffer and incubated in the wells for

30 minutes. Standard calibrators, calibrated against purified MRP8/14 from human granulocytes by the manufacturer are included at concentrations of 4, 12, 40, 120, and 240 ng/ml. MRP8/14 present binds to the specific capture antibody. A second monoclonal detection antibody conjugated to horseradish peroxidase (HRP) detects the MRP8/14 molecules bound to the monoclonal antibody coated onto the plate after a washing step. After 30 minutes incubation and a further washing step, tetramethylbenzidine (TMB, in citrate buffer with H_2O_2) is added for 20 minutes, followed by a stopping reaction (0.25M sulphuric acid). The absorption of the resulting coloured product is measured at 450 nm. Manufacturer quality control samples, as well as in-house derived control samples, were included to assure assay performance. The final concentration of MRP8/14 was calculated by averaging the results obtained at 1:100 and 1:400 (Biotek ELx808 absorbance microplate reader, Biotek Instruments Inc., USA, using Gen5 Reader control software).

Intra-assay variability was in keeping with the manufacturer's stated performance (4% reported by Buhlmann versus 5% in our lab). Inter-assay variability was slightly higher than the manufacturer's stated performance, but within limits considered to be acceptable for this type of assay as performed in a clinical laboratory (6% reported by Buhlmann versus 11% in our lab). Linearity of the assay was established by running a quality control sample serially diluted and measuring percentage recovery. Performance was in keeping with manufacturer's performance (range 85%-110% reported by Buhlmann, 96%-110% in our lab). To establish stability of MRP8/14 in blood serum, a sample from a healthy adult control was kept at room temperature and processed on five consecutive days, with all samples being run on the same plate. The criterion for variability was initially set at <10%. The value obtained was 12%- although this was outside the target set, it was accepted as being in keeping with the expected performance for this type of assay in a clinical lab. For the purposes of validation, the comparison samples were diluted until a numerical result was obtained. Post-validation, samples with values of greater than 15,000ng/ml are routinely reported by the clinical lab as >15,000ng/ml.

To establish whether a paediatric reference range was required, MRP8/14 was measured in 63 healthy children and 49 healthy adults. No significant difference was observed. MRP8/14 was measured in 10 serum samples from healthy children from 2 age groups (≤5y and >5y). The data were compared between the 2 groups. No significant difference between the groups and no correlation between age and MRP8/14 was observed (Spearman r=L0.067), indicating that an age specific reference range was not required.

To ensure correlation of results with a previously established method, duplicate aliquots of JIA serum samples taken prior to starting methotrexate were analysed by an in-house assay in Munster. Figure S1 shows that the assays have good correlation (Spearman r=0.967), although numeric values in the commercial Buhlmann ELISA were five times higher than in the Munster in-house assay, as previously published [12]. In this study, the cut-off for risk of flare was determined to be 690ng/ml using the Munster assay, so for the Buhlmann assay the cut-off was set at approximately five times this i.e. 4000 ng/ml [12].

Figure S1: Validation data of the commercial Buhlmann MRP8/14 ELISA against an established in-house MRP8/14 ELISA (n=107).

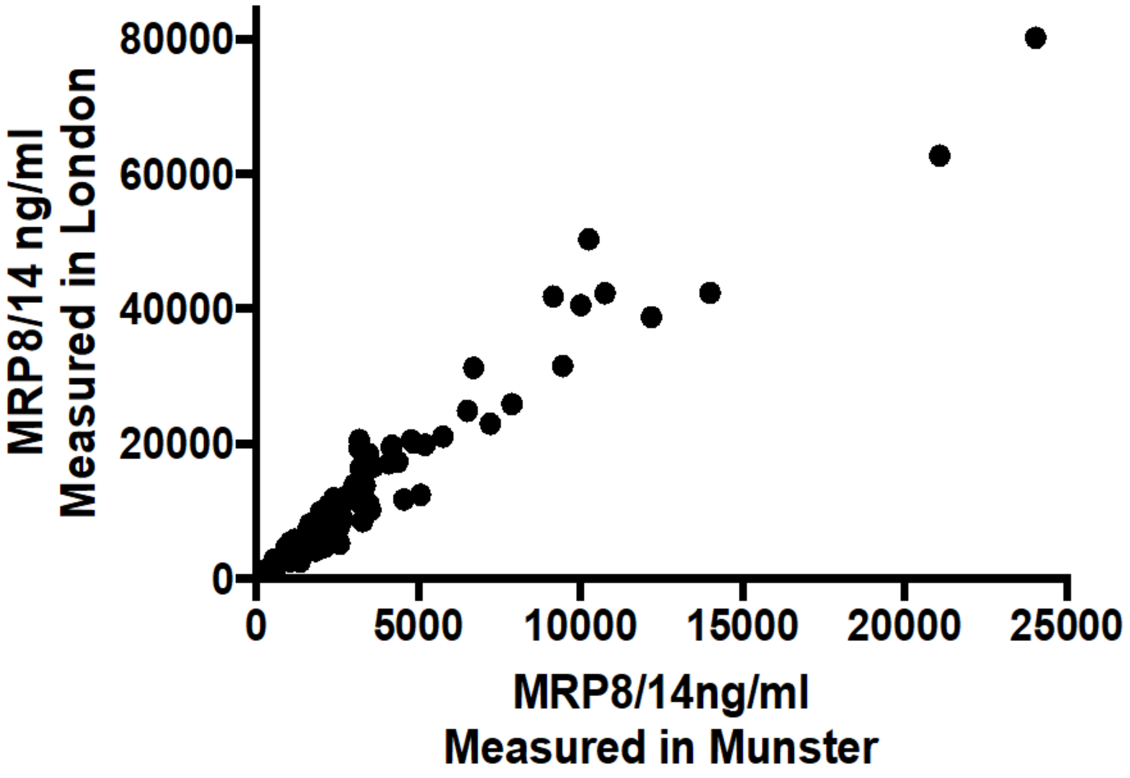

Table S1: Survey of clinicians regarding MRP8/14 and clinical decision-making

<table>
<tr><td colspan="60">1) What is your job role?</td></tr>
<tr><td colspan="20">Consultant</td><td colspan="20">Clinical Academic</td><td colspan="20">Clinical Nurse Specialist</td></tr>
<tr><td colspan="20">3</td><td colspan="20">1</td><td colspan="20">1</td></tr>
<tr><td colspan="60"></td></tr>
<tr><td colspan="60">2) How often do you order MRP8/14 in your clinical practise?</td></tr>
<tr><td colspan="30">Weekly</td><td colspan="30">Monthly</td></tr>
<tr><td colspan="30">2</td><td colspan="30">3</td></tr>
<tr><td colspan="60"></td></tr>
<tr><td colspan="60">3) What are your reasons for ordering MRP8/14? (multiple allowed)</td></tr>
<tr><td colspan="15">Stopping medication</td><td colspan="15">Starting or escalating medication</td><td colspan="15">Checking response to therapy</td><td colspan="15">Assessing for flare on or off therapy</td></tr>
<tr><td colspan="15">5</td><td colspan="15">3</td><td colspan="15">1</td><td colspan="15">1</td></tr>
<tr><td colspan="60"></td></tr>
<tr><td colspan="60">4) What medication do you use MRP 8/14 testing for? (multiple allowed)</td></tr>
<tr><td colspan="12">MTX</td><td colspan="12">Etanercept</td><td colspan="12">Adalimumab</td><td colspan="12">Infliximab</td><td colspan="12">Azathioprine and mycophenolate</td></tr>
<tr><td colspan="12">5</td><td colspan="12">2</td><td colspan="12">2</td><td colspan="12">1</td><td colspan="12">1</td></tr>
<tr><td colspan="60"></td></tr>
<tr><td colspan="60">5) If your patient is on MTX and a biologic and has a low MRP 8/14, which do you stop?</td></tr>
<tr><td colspan="20">Methotrexate</td><td colspan="40"></td></tr>
</table>

5		
**6) Does the current wait of 2-4 weeks for MRP 8/14 results influence your decision?**		
Yes, waiting for MRP 8/14 results delays my decision	*No, MRP 8/14 results come at a reasonable time so I make my decision with the result*	
2	3	
**7) At what MRP 8/14 result would you consider stopping medications?**		
Low (<4,000 ng/ml)		
5		
**8) Do you think MRP 8/14 testing has improved clinical outcome when deciding to stop medications?**		
Yes	*No*	
3	2	
Comments: “parents and patients have to be informed that it isn’t a guarantee that their child wont flare but is one of the factors used in the decision to stop treatment”		
**9) Would you recommend MRP 8/14 testing to other paediatric rheumatology departments?**		
Yes	*No*	
4	1	
Comments: “probably best to use a combination of biomarkers to assess the risk better”		
**10) How do you feel more research into the use of MRP testing in rheumatology would be beneficial? (multiple allowed)**		
Improving confidence in predicting response to treatment	*Improving confidence in predicting flare*	*Acting as a predictor of disease activity.*
4	3	2

R Code for Cox Proportional Hazards

```
# install package so R can read excel files
> install.packages("readxl")

# load required packages and read excel containing raw data, replace "_____" with file path to excel document.
> library(survival)
> library(readxl)
> x <- read_xlsx("~/_____")

# make it a normal data frame, view and check for errors
> x <- as.data.frame(x)
> colnames(x) <- make.names(colnames(x))
> head(x)
> warnings()

# set follow up months as time and create a survival analysis object
> x$time <- x$Fup
> x$s <- Surv(time=x$time, event=x$Flare=="Yes")

# set the factor as MTX outcome (stop/"yes", didn't stop/"no", "wean") and create table to see number in each group
> x$mtx <- factor(x$MTX.stop)
> table(x$mtx)

# fit the cox regression against whether patient stopped MTX
> m <- coxph(s ~ mtx,data=x)
```

```
> m
Call:
coxph(formula = s ~ mtx, data = x)
>

# fit new cox regression only looking at MTX outcome is stop/“yes” or didn’t stop/“no”
> x0 <- subset(x, MTX.stop %in% c("Yes","No"))
> x0mtx <- factor(x$MTX.stop)
> m <- coxph(s ~ mtx, data=x0)
> m

# fit new cox regression using mrp result as numeric
> x$MRP <- as.numeric(x$MRP.Result..GOSH.)
> m <- coxph(s ~ MRP,data=x)
> m

# re-read excel containing raw data,  replace “_____” with file path to excel document
> x <- read_xlsx("~/_____")
# make it a normal data frame, view and check for errors
> x <- as.data.frame(x)
> colnames(x) <- make.names(colnames(x))
> head(x)

# set follow up months as time and create a survival analysis object
> x$time <- x$Fup
> x$s <- Surv(time=x$time,event=x$Flare=="Yes")
```

```
# set the factor as Wallace criteria (is patient in clinically inactive disease “yes” or “no”) and create table to see number in each group
> x$wallace <- factor(x$Wallace.Met)
> table(x$wallace)

 # fit the cox regression against whether patient had clinically inactive disease or not
> m <- coxph(s ~ wallace, data=x)
> m

# fit new cox regression using mrp result as numeric in only patients who had clinically inactive disease/Wallace “Yes”
> x$MRP <- as.numeric(x$MRP.Result..GOSH.)
>xwallace <- subset(x,Wallace.Met==”Yes”)
>m <-coxph(s ~ MRP, data=xwallace)
>m
>
```
